# Effects of In Ovo Injection of Zinc or Diet Supplementation of Zinc on Performance, Serum Biochemical Profiles, and Meat Quality in Broilers

**DOI:** 10.3390/ani12050630

**Published:** 2022-03-02

**Authors:** Hee-Jin Kim, Hwan-Ku Kang

**Affiliations:** Poultry Research Institute, National Institute of Animal Science, Rural Development Administration, Pyeongchang 25342, Korea; khj0175@korea.kr

**Keywords:** broiler, immunoglobulin, in ovo injected, body weight, zinc, fatty acid

## Abstract

**Simple Summary:**

Zinc (Zn) is known as an essential trace mineral that plays a key role in metabolic processes such as synthesis, stability, and catalytic activity of many proteins in broilers. In ovo technology provides an alternative for delivering nutrient compounds to the embryo prior to hatching by injecting small amounts of material into a broiler egg during hatching. In this study, the in ovo injection of Zn and dietary supplementation of Zn were evaluated on their effects on growth performance, serum biochemical profiles, and meat quality in broilers. In ovo injection and Zn supplementation (i.e., 200 mg/kg) increased (*p* < 0.05) body weight and immunoglobulin G (IgG) compared to broilers fed with the control diet. In ovo injection group decreased (*p* < 0.05) feed conversion ratios compared to broilers fed with the no injection group. Our results indicated that broilers fed with a diet supplemented with 200 mg/kg of Zn could ameliorate growth performance, immune responses, and increase the unsaturated fatty acids of breast meat. The findings of this result highlighted that the diet supplemented with Zn has the effect to improve performance, blood immune response, and fatty acid profile of breast meat.

**Abstract:**

This study investigated the main effects of the in ovo injection of inorganic zinc (Zn) or diet supplementation of Zn on performance, serum biochemical profiles, and breast meat quality in broilers. A total of 480 one-day-old broilers (Ross 308) were randomly divided into four groups: the control (Con, noninjected and basal diet), in ovo (injected 60 mg Zn/egg at 18 embryonic days of incubation and basal diet), Zn100 (noninjected and basal diet with Zn (100 mg/kg) for 35 days), and Zn200 (noninjected and basal diet with Zn (200 mg/kg) for 35 days) groups. The dietary supplementation of Zn increased feed intake (2860.42–2861.08 g), weight (1975.06–1985.25 g), and weight gain (1936.36–1946.53 g) compared to Con (2785.74, 1891.38, and 1852.62 g, respectively) after five weeks of age. No significant difference was found in biochemical parameters and leukocyte and erythrocyte levels in the blood among the four different groups. In ovo injected or supplemental Zn (100 and 200 mg/kg) increased IgG in the blood of broilers. Zn200 increased polyunsaturated fatty acids, and saturated fatty acid contents were reduced in breast meat compared with Con. In conclusion, Zn supplementation at 200 mg/kg could improve the weight, feed intake, blood immune response, and fatty acid profile of breast meat.

## 1. Introduction

Among minerals regularly used in chicken, zinc (Zn) performs a specific activity in broilers as a result of its metabolic connection to skeletal integrity and growth [1,2]. Additionally, Zn serves as an antioxidant supplement that protects cells from oxidative damage [3]. Frequently, higher contents of Zn are supplemented in feed for improving immune responses in broilers because Zn mediates the continuous activity of superoxide dismutase (SOD), which is essential for the macrophage and heterophil integrity of broilers [4]. In ovo Zn injections and dietary Zn supplementation improved Zn and copper expression levels containing SOD and metallothionein as free radical scavenging enzymes in broiler tissues [5]. However, the ingestion of excess mineral content affects the bioavailability of birds and may have antagonistic effects on other nutrients in broilers [6]. Excessive Zn supplementation has an adverse effect on the bioavailability of other minerals [7]. Some experiments observed increased weight and positive effects when dietary 80–180 mg/kg Zn concentrations were present [8,9]. However, another experiment did not affect the weight gain of broiler when dietary 160 mg/kg Zn concentrations were present [10]. Based on those findings, two supplemental Zn concentrations (100 and 200 mg/kg) were chosen in the current study.

Production reductions in poultry because of reduced immunity and poor health are the main interest of many poultry companies. To cut these losses, intervention strategies that include nutritional supplementation before and after hatching were developed [11]. In the poultry industry, in ovo vaccination is used to control diseases. However, many researchers have applied the technology of in ovo injection to add nutrients such as vitamins [12], amino acids [13], minerals [11], and carbohydrates [14]. The expected improvements in response to this type of supplement include hatchability, bone development, posthatch performance, and immunity [11]. The egg yolk is the central storage part of nutrients for the embryo, but Zn is mostly depleted in the latter embryonic step [15]. In ovo injection of Zn, manganese, Cu, and iron enhances mineral levels as the consumption of embryonic minerals increases in the yolk [15]. The other studies suggested that the in ovo injection of carbohydrates [16], gava [17], and arginine [18] at a concentration of about 0.1% of egg weight improved broiler health and productivity.

However, few reports have been conducted on the effect of in ovo injected or dietary Zn on performance, blood biochemical profiles (such as serum glucose, protein, lipid, and mineral), and broiler breast meat quality. This study evaluated the effects of in ovo injection or supplementation of Zn on the performance, serum biochemical profiles, and breast meat quality of broilers.

## 2. Materials and Methods

The protocol for this experiment was examined and approved by the Institutional Animal Care and Welfare Committee of the National Institute of Animal Science, Rural Development Administration, Republic of Korea (2019–364).

### 2.1. Birds and Experimental Design

A total of 480 one-day-old broilers (Ross 308) were randomly allocated into one of four equal treatments to provide four replications. The complete experiment was conducted according to a randomized design of treatments. The control group was neither injected nor fed with Zn supplementation (Con). The in ovo group was injected with Zn (60 mg/egg). The remaining two groups were provided with basal diets containing Zn at either 100 or 200 mg/kg levels. Zn used for feed, and in ovo injection was served as the inorganic Zn source. All eggs were collected from the same commercial broiler breeder company and transported to the Poultry Research Institute at the same time. The eggs were set in an incubator with a temperature of 37.8 °C and relative humidity of 60%. Infertile eggs were removed on the 18th day. The eggs were placed back in the incubator with a hatcher temperature of 37.8 °C and relative humidity of 60% until the 21th day. The stock solutions were diluted with distilled water (DW) to make injection solutions of Zn containing 600 mg/mL. On the eggs, 0.1 mL of Zn solution was injected to provide doses of 600 mg Zn/mL. At 18 embryonic days of incubation, the treatment solutions were injected into the 400 eggs using Gerstel MPS2 (GERSTEL, Mülheim, Germany). The equal volume of 0.1 mL was injected automatically into the air chamber by using self-refilling syringes. After injection, each hole was sealed using wax, and egg incubation was continued until hatching. Other groups were not injected with Zn. Among the hatched chickens, 480 males were randomly chosen (120 birds for each group, *n* = 30 birds in each 4 pen). The basal corn–soybean meal diets in both starter (0 to 21 days) and grower (22 to 35 days) periods were formulated using mineral premix (Zn-free) (Table 1). The diets were fed in either mash (starter) or crumble (grower) form to broilers. The basic diet was provided as a commercial feed without added zinc. Measured quantities of inorganic Zn were included in the basal diet to confirm Zn supplementation at 100 and 200 mg/kg. Initially, the basal diet was provided for the chicks for seven days. Experimental diets were provided on an ad libitum basis from 8 to 35 days of age. An intermittent lighting schedule comprising 3:1 h (light:dark) cycles was practiced from 8 to 35 days of age.

### 2.2. Performance (Weight Gain and Feed Efficiency)

All individual birds were weighed on day 1, 7, 21, and 35. Feed intakes of broilers were measured on day 21 and 35, and the feed conversion ratio (FCR) was calculated as the ratio between the feed intake and the gained body weight.

### 2.3. Haematological Analysis

Blood samples were collected and bled via wing vein at the age of 35 days before slaughtering. Blood samples from the jugular vein of each broiler were collected using non-EDTA (ethylenediaminetetraacetic acid)-treated and EDTA-treated Becton vacutainer tubes (Becton Dickinson, Franklin Lakes, NJ, USA). Serum was obtained by centrifuging broiler blood samples at 3000× *g* for 10 min, after which it was stored at −75 °C. The total cholesterol (T. chol), glucose (GLU), aspartate aminotransferase (AST), albumin (ALB), triglyceride (TG), total protein (TP), and alanine aminotransferase (ALT) in serum were quantified by using the Chemistry Analyzer Chemistry System (AU480 Chemistry Analyzer, Beckman Coulter Inc., Brea, CA, USA). The broiler’s whole blood samples were immediately utilized for hematological analysis (Leukocytes and erythrocytes) using the Hemavet Multi-Species Hematology System (Drew Scientific Inc., Oxford, CT, USA).

### 2.4. Immune Response

Immunoglobulin G (IgG) activity was assayed using a Chicken IgG ELISA Kit (MyBioSource, Inc., San Diego, CA, USA). Absorbance was recorded using a microplate reader at 450 nm (Epoch 2; BioTek Instruments, Inc., Winooski, VT, USA).

### 2.5. Meat Quality

The forty broilers were randomly chosen and slaughtered by cervical dislocation after the experiments. After bleeding for 5 min, the birds were scalded in a hot water bath before feathers were plucked and eviscerated, and breast meat samples were collected. Breast samples from the left side (140~180 g) were used for measuring the proximate composition (moisture, crude protein, crude fat, and crude ash), pH, water holding capacity (WHC), and fatty acid, while samples from the right side were used for measuring cooking loss and shear force. Moisture, crude protein, crude fat, and crude ash of breast meat were measured according to the Association of Official Analytical Chemists methods [19]. The pH value of the breast meat was measured as follows: 10 g of breast meat was homogenized with 90 mL of DW for 30 s using a homogenizer according to Kim et al. [20], and the pH value of the homogenates was measured using a pH meter (Model 340, Mettler-Toledo GmbH, Schwerzenbach, Switzerland). WHC, cooking loss, and shear force were evaluated according to Kim et al. [20].

### 2.6. Fatty Acid Composition of Meat

The fatty acid of breast meat was evaluated according to Kim et al.’s methods [20]. After 24 h of cooling at a temperature of 4 °C, breast meat was stored in a freezer at a temperature of −20 °C until analyses. Breast meat was homogenized by grinding using a mixer. The lipids were extracted with the addition of butylated hydroxyanisole and Folch solution (1:2 mixture of methyl alcohol and chloroform) from breast meat. The homogenates were vortexed with 0.88% KCl and separated by two layers. The lower layer (lipid layer) was condensed with N2 gas. A lipid sample was mixed with 0.5 N NaOH (in methyl alcohol) and heated to 100 °C using a water bath for 5 min. The mixture was added with 10% boron trifluoride, heated to 100 °C for 2 min, and mixed with saturated NaCl and iso-octane. The iso-octane extract was analyzed by using a gas chromatograph (Agilent 6890N, Agilent Technologies, Wilmington, DE, USA). An Omega wax 250 capillary column (Supelco, Bellefonte, PA, USA), with 30 m × 0.25 mm × 0.25 μm film thickness, was used for fatty acid composition analysis.

### 2.7. Statistical Analysis

The effects of in ovo injected and dietary zinc were estimated with one-way analysis of variance using the general linear model of the SAS program (ver. 9.4, SAS Institute Inc., Cary, NC, USA). Mean separation was conducted using Tukey’s multiple range test, and statistical significance was regarded at *p* < 0.05.

## 3. Results and Discussion

### 3.1. Performance

The effects of in ovo and supplemental Zn on broiler performance are shown in Table 2. At 1–21 days of age, weight gain, feed intake, and FCR were not affected by treatments. At day 35, a higher weight was observed in the in ovo injected (1987 g) and supplemental Zn treatments (1975–1975 g) compared with Con (1891 g) (*p* < 0.05). However, weight gain, feed intake, and FCR were not affected by all treatments at 22-35 days of age. At 1–35 days of age, higher weight gain (1936–1946 g) and feed intake (2860–2861 g) were observed in supplemental Zn treatments (100 mg and 200 mg/kg) compared with Con (1852 and 2785 g, respectively). However, in ovo injected Zn (1.46) treatments showed lower FCR compared with Con (1.50).

### 3.2. Serum Biochemistry Characteristics

The effects of in ovo injected and supplemental Zn on serum biochemistry characteristics are shown in Table 3. T. chol (172.82–195.90 mg/dL), GLU (279.70–294.77 mg/dL), AST (477.20–588.20 U/L), ALB (1.21–1.38 g/dL), TG (98.38–111.08 mg/dL), TP (2.50–3.17 g/dL), and ALT (3.48–3.85 U/L) were not affected by the treatments.

### 3.3. Haematological Analysis and IgG of Blood

**Figure 1:**
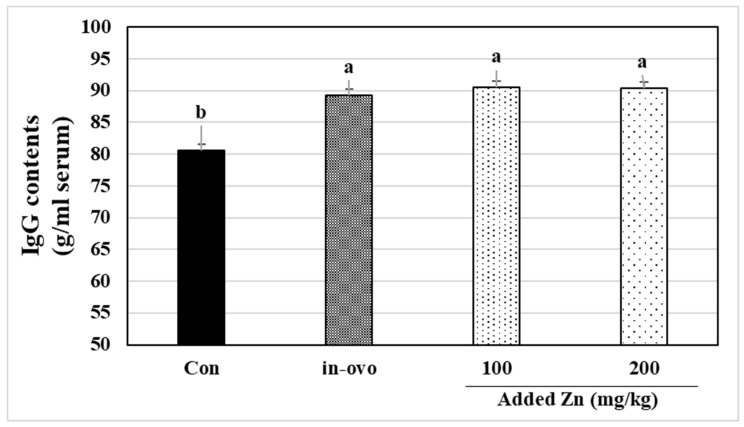
Effect of in ovo injected and supplemental Zn on IgG of blood in broilers at 35 days of age. ^a,b^ Means in treatment with different superscripts are significantly different (*p* < 0.05).

The effects of in ovo injected and supplemental Zn on leukocytes and erythrocytes are shown in Table 4. No significant differences were found in leukocyte, red blood cells, hemoglobulin (Hb), hematocrit (HCT), mean corpuscular hemoglobulin, and mean corpuscular hemoglobulin concentration levels in broiler blood. However, significant differences were found in the mean corpuscular volume. The effects of in ovo injected and supplemental Zn on IgG in broiler blood are shown in Figure 1.

### 3.4. Meat Quality

The effects of in ovo injected and supplemental Zn on breast meat quality are shown in Table 5. The effect of in ovo injected and supplemental Zn (100 and 200 mg/kg) was not significant for proximate composition (moisture, crude protein, crude fat, and crude ash), cooking loss, and shear force. The lower pH was observed in supplemental 200 mg/kg Zn treatments compared with Con (*p* < 0.05).

### 3.5. Fatty Acid Composition of Breast Meat

The effects of in ovo injected and supplemental Zn on the fatty acid composition of breast meat are shown in Table 6. The three main fatty acids in breast meat were oleic acid (45.99–45.10%), palmitic acid (23.13–23.72%), and linoleic acid (14.86–15.65%). The supplemental Zn (200 mg/kg) group showed lower contents of saturated fatty acid (SFA) than the Con group, and the supplemental Zn (200 mg/kg) group showed higher contents of unsaturated fatty acid (USFA) and polyunsaturated fatty acid (PUFA) than Con (*p* < 0.05).

## 4. Discussion

The in ovo injection of some nutrients, such as minerals and vitamins, might reduce the harmful effects of stress, which could have a positive effect on posthatch health and growth performances of broilers [21]. Zn positively influences feed usage by its inclusion in the metabolism of proteins, lipids, and carbohydrates [22]. The findings in this research are in contrast to the reports of Pimentel et al. [23], who reported that supplemental Zn (28–88 mg/kg) had no significant influence on body weight, feed intake, and FCR of broilers. Jahanian et al. [24] reported that zinc-methionine (40, 80 and 120 mg/kg) supplementation had no beneficial impacts on body weight neither in the starter nor in overall periods. The possible account for this difference could be the differences in Zn source contents and physicochemical properties. Hudson et al. [10] (160 mg/kg, zinc sulphate or zinc-amino acid complex) and Huang et al. (20–140 mg/kg, zinc sulfate heptahydrate) [9] reported that Zn sources influenced the weight gain of broilers. According to Zakaria et al. [25], the performance response of broilers is not different between 80 (inorganic Zn) and 122 (inorganic Zn + zinc-methionine) mg/kg. Saenmahayak et al. [26] found that the dietary supplementation of Zn, regardless of its sources and form over 40 mg/kg of diet, had no effect on the growth rate of broilers. Jose et al. [27] reported that in ovo injected 0.25 and 0.50 mg/egg Zn (Zn sulphate, Zn methionine, and nano Zn oxide) did not increase the growth performances of posthatch broilers. However, in this research, higher concentrations of in ovo injection Zn (60 mg/egg) presented overall better weight gain. The progressive addition of an inorganic form (0–1500 ppm) [28], an organic form (0–60 ppm) [29], or a combination form of the diet did not influence the FCR of broilers. Results showed that Zn in ovo injection and supplementation did not affect FCR, but Zn in ovo injection and supplementation (100 and 200 mg/kg) showed significantly better body weight than Con.

Serum biochemistry parameters are important indicators of physiological and pathological changes, nutrition, and normal health status occurring in broilers [30]. Moreover, Zn is an important element in the synthesis, secretion, and storage of insulin and is related to glucose contents in the blood [31]. This agrees with Zakaria et al. [25], who reported that the glucose, cholesterol, phosphate, TP, and albumin of serum were not influenced by Zn supplementation. By contrast, Uyanik et al. [32] found that Zn supplementation reduced the glucose content of serum, which has been found to be related to zinc and insulin metabolism. Similarly to our results, Lu and Combs [33] reported that Zn supplementation (ZnO, 87~1060 mg/kg) in broiler chick did not influence glucose. Christianson [34] reported that Zn has a critical function in body protein turnover and affects protein metabolic wastes and its protein degradation. However, there was no significant difference in the TP contents of serum in this study.

Hematological indices of blood provide major information about broilers’ health [35]. Zn is a major factor in all aspects of immunity [36] and plays an essential role in cells involved in immune responses [37,38]. Sridhar et al. [39] reported that WBC, RBC, Hb, and LY in broiler chicks fed with lower concentrates (10–20 ppm) of Zn from organic sources was comparable with those on 40 ppm Zn from an inorganic source. This agrees with Kwiecień et al. [35], who found that WBC, LY, RBC, MCH, Hb, and He were not affected by ZnO supplementation (100 mg/kg). Hassan [40] found that WBC, LY, MO, BA, RBC, HCT, MCH, and MCHC is not different in ovo injection zinc (15 ppm/egg) treatment and uninjected treatment. Lebacq-Verheyden et al. [41] found that IgG accounts for the major portion of immunoglobulins in chicken blood, followed by IgA and IgM. The lowest IgG was shown in the control treatment broiler compared to in ovo injected and supplemental Zn treatments. Bartlett and Smith [42] reported that broilers that received supplemental Zn (68 and 181 mg) had a higher IgG, showing that Zn significantly affects immune response in birds. However, there is a lack of sufficient evidence on the use of these in ovo injection and supplementation of zinc and their effects on the immune system.

Sensory meat quality for the broiler is mainly reflected in pH, WHC, and shear force (tenderness). Meat quality, such as pH, shear force, WHC, shelf life, and protein content, are essential for meat products [43]. Liu et al. [8] and Salim et al. [44] reported that shear force in meat was not different from the supplemental Zn level (0–180 mg and 0–25 ppm, respectively). In broilers, many dietary factors affect meat tenderness (shear force) [44,45]. The results from our study are similar to those reported by Saenmahayak et al. [46], where Zn supplementation had no effect on the cooking loss and WHC of meat. Zn sources on meat quality of broilers have limited information on the effects, and experiments are needed to further elucidate the effect of Zn sources on the meat quality of broilers [47]. The pH of meat affects the rate of microbial spoilage [48]. Higher meat pH facilitates the growth of spoilage bacteria than low meat pH when stored under the same storage conditions [49]. Therefore, the breast meat of supplemental Zn (200 mg/kg) groups is expected to increase shelf life on aerobic storage. However, more experiments are needed to determine the effect of in ovo injected and supplemental Zn on broiler meat quality.

Our results were similar to those of Nkukwana et al. [50], who observed that the three major fatty acids in breast meat were oleic acid (28.0–33.6%), palmitic acid (21.6–25.3%), and linoleic acid (16.3–21.3%). High levels of long-chain SFA (except stearic acid) intake were implicated in increasing blood cholesterol levels in humans [51]. Therefore, we could speculate that dietary Zn (200 mg/kg) might increase the USFA and PUFA of meat. More experiments are needed to elucidate the effect of Zn on the fatty acid of meat.

## 5. Conclusions

Dietary supplementation of Zn at the level of 200 mg/kg would improve both immune response (IgG) of broilers and PUFA of chicken breast meat. Further research may be warranted to investigate the mechanism of transport and absorption of Zn in the broiler.

## Figures and Tables

**Table 1:** Composition and nutrient content of experimental diets (as-fed basis).

Parameters	Starter Diet (0 to 21 Days)	Grower Diet (22 to 35 Days)
Ingredients, %		
Corn, US No. 3	51.92	54.67
Soybean meal-44% CP	28.10	23.00
Wheat meal	5.00	10.00
Corn gluten	3.84	2.01
Fish meal	4.00	3.50
Tallow	3.50	3.50
Dicalcium phosphate	1.86	1.59
Limestone	1.00	1.00
Sodium chloride	0.22	0.25
Choline-50%	0.06	0.04
Methionine-99%	0.11	0.11
Lysine-78%	0.14	0.11
Vitamin and mineral premix ^1^	0.24	0.22
Total	100.00	100.00
Nutrient content		
MEn, kcal/kg	3100	3150
CP, %	22.00	19.00
Ca, %	1.00	0.92
Available p, %	0.51	0.45
Met + Cys, %	0.87	0.75

^1^ Provided per kilogram of the complete diet: vitamin A (from vitamin Aacetate), 12,500 IU; vitamin D3, 2500 IU; vitamin E (from DL-a-toco-pheryl acetate), 20 IU; vitamin K3, 2 mg; vitamin B1, 2 mg; vitamin B2, 5 mg; vitamin B6, 3 mg; vitamin B12, 18 μg; calcium pantothenate, 8 mg; folic acid, 1 mg; biotin, 50 μg; niacin, 24 mg; Fe (as FeSO4 7H2O), 40 mg; Cu (as CuSO4 H2O), 8 mg; Zn (as ZnSO4 H2O), 60 mg; Mn (asMnSO4 H2O), 90 mg; Mg (MgO) as 1500 mg.

**Table 2:** Effect of in ovo injected and supplemental Zn on growth performance in broilers at 35 days of age.

Parameters	Con	In Ovo	Added Zn (mg/kg)		SEM ^1^	*p*-Value
			100	200		
Initial weight (g)	38.73	38.70	38.73	38.73	0.035	0.911
Starter (day 1–21)						
Weight (g)	984	991	990	1018	10.8	0.18
Weight gain (g)	945	953	951	979	10.7	0.18
Feed intake (g)	839	888	902	892	17.1	0.09
FCR	0.89	0.93	0.95	0.91	0.02	0.09
Grower (day 22–35)						
Weight (g)	1891 ^b^	1987 ^a^	1975 ^a^	1985 ^a^	24.59	<0.05
Weight gain (g)	907	998	983	968	31.30	0.24
Feed intake (g)	1946	1952	1972	1969	13.27	0.47
FCR	2.17	1.96	2.01	2.04	0.06	0.18
Overall (day 1–35)						
Weight gain (g)	1853 ^b^	1949 ^a^	1936 ^a^	1946 ^a^	9.92	<0001
Feed intake (g)	2786 ^b^	2854 ^ab^	2860 ^a^	2861 ^a^	16.53	<0.05
FCR	1.50 ^a^	1.46 ^b^	1.48 ^ab^	1.47 ^ab^	0.01	<0.05

^1^ SEM, standard error of means. ^a,b^ Means in the same rows with different superscripts are significantly different (*p* < 0.05).

**Table 3:** Effect of in ovo injected and supplemental Zn on blood biochemistry characteristics in broilers at 35 days of age.

Parameters	Con	In Ovo	Added Zn (mg/kg)		SEM ^1^	*p*-Values
			100	200		
T. chol (mg/dL) ^2^	189	196	178	173	9.27	0.59
GLU (mg/dL)	295	290	285	280	9.67	0.72
AST (U/L)	588	477	575	555	54.3	0.51
ALB (g/dL)	1.37	1.38	1.21	1.28	0.07	0.32
TG (mg/dL)	125.5	111.1	98.4	99.3	12.9	0.12
TP (g/dL)	3.17	2.50	3.00	2.57	0.39	0.58
ALT (U/L)	3.61	3.85	3.48	3.51	0.21	0.59

^1^ SEM, standard error of means. ^2^ T. chol, total cholesterol; GLU, glucose; AST, aspartate aminotransferase; ALB, albumin; TG, triglyceride; TP, total protein; ALT, alanine aminotransferase; IP, inorganic phosphorus.

**Table 4:** Effect of in ovo injected and supplemental Zn on leukocytes and erythrocytes in broilers at 35 days of age.

Parameters	Con	In Ovo	Added Zn (mg/kg)		SEM ^1^	*p*-Values
			100	200		
Leukocytes						
WBC (K/μL) ^2^	17.8	19.9	20.2	20.4	1.65	0.65
HE (K/μL)	4.86	5.86	6.30	5.99	0.77	0.59
LY (K/μL)	10.4	10.8	10.6	11.2	0.52	0.68
H/L	0.46	0.53	0.59	0.52	0.05	0.39
MO (K/μL)	1.78	2.20	2.00	2.14	0.20	0.47
EO (K/μL)	0.57	0.78	0.97	0.80	0.18	0.50
BA (K/μL)	0.17	0.26	0.37	0.24	0.09	0.53
Erythrocyte						
RBC (K/μL)	2.23	2.35	2.47	2.38	0.13	0.63
Hb	7.78	8.18	8.64	8.24	0.46	0.63
HCT (%)	22.9	24.4	27.6	25.1	1.49	0.20
MCH (g/dL)	34.9	34.7	34.9	34.1	0.64	0.81
MCHC (g/dL)	33.9	33.6	31.4	28.6	2.46	0.41

^1^ SEM, standard error of means. ^2^ WBC, white blood cells; HE, heterophils; LY, lymphocytes; H/L, Heterophil: lymphocytes; Mo, monocytes; EO, eosinophils; BA, basophils; RBC, red blood cells; Hb, hemoglobulin; HCT, hematocrit; MCH, mean corpuscular hemoglobulin; MCHC, mean corpuscular hemoglobulin concentration.

**Table 5:** Effect of in ovo injected and supplemental Zn breast meat quality in broilers at 35 days of age.

Parameters	Con	In Ovo	Added Zn (mg/kg)		SEM ^1^	*p*-Value
			100	200		
Proximate composition (%)						
Moisture	75.9	76.0	74.6	75.0	0.26	0.05
Crude protein	22.3	22.0	23.0	22.87	0.50	0.46
Crude fat	2.10	2.24	2.61	2.14	0.31	0.65
Crude ash	1.18	1.19	1.23	1.26	0.04	0.51
pH	5.92 ^ab^	6.01 ^a^	5.91 ^b^	5.81 ^c^	0.03	<0.05
Cooking lose (%)	18.8	18.0	17.2	15.87	0.86	0.12
WHC ^2^ (%)	62.1	61.3	61.7	62.35	0.85	0.84
Shear force (N)	25.3	22.9	26.0	21.10	1.83	0.25

^1^ SEM, standard error of means. ^2^ WHC, water holding capacity. ^a–c^ Means in the same rows with different superscripts are significantly different (*p* < 0.05).

**Table 6:** Effect of in ovo injected and supplemental Zn on fatty acid composition of breast meat in broilers at 35 days of age.

Parameters	Con	In Ovo	Added Zn (mg/kg)		SEM ^1^	*p*-Value
			100	200		
Myristic acid (C14:0)	0.98	0.94	0.97	0.94	0.01	0.16
Palmitic acid (C16:0)	23.7	23.4	23.6	23.1	0.16	0.08
Palmitoleic acid (C16:ln7)	6.20 ^ab^	6.08 ^b^	6.5 ^a^	6.36 ^ab^	0.11	<0.05
Stearic acid (C18:0)	6.40	6.38	6.3	6.10	0.10	0.15
Oleic acid (C18:ln9)	45.9	46.0	45.1	45.6	0.26	0.10
Linoleic acid (C18:2n6)	14.9	15.2	15.2	15.6	0.23	0.14
γ-Linoleic acid (C18:3n6)	0.23 ^a^	0.19 ^b^	0.19 ^b^	0.20 ^ab^	0.01	0.01
Linolenic acid (C18:3n3)	0.69	0.75	0.76	0.77	0.04	0.36
Eicosenoic acid (C20:ln9)	0.63	0.64	0.63	0.63	0.01	0.86
Arachidonic acid (C20:4n6)	0.37 ^c^	0.41 ^c^	0.71 ^a^	0.57 ^b^	0.04	<0001
SFA ^2^	31.1 ^a^	30.7 ^ab^	30.9 ^ab^	30.2 ^b^	0.19	<0.05
USFA	68.9 ^b^	69.2 ^ab^	69.1 ^ab^	69.8 ^a^	0.19	<0.05
MUFA	52.7	52.7	52.3	52.6	0.25	0.51
PUFA	16.1 ^b^	16.5 ^b^	16.9 ^ab^	17.2 ^a^	0.24	<0.05

^1^ SEM, standard error of means. ^2^ SFA, saturated fatty acid; USFA, unsaturated fatty acid: MUFA, monounsaturated fatty acid; PUFA, polyunsaturated fatty acid. ^a–c^ Means in the same rows with different superscripts are significantly different (*p* < 0.05).

## Data Availability

Not applicable.
